# DNA Elements Reducing Transcriptional Gene Silencing Revealed by a Novel Screening Strategy

**DOI:** 10.1371/journal.pone.0054670

**Published:** 2013-01-30

**Authors:** Naoki Kishimoto, Jun-ichi Nagai, Takehito Kinoshita, Keiichiro Ueno, Yuko Ohashi, Ichiro Mitsuhara

**Affiliations:** 1 Agrogenomics Research Center, National Institute of Agrobiological Sciences (NIAS), Tsukuba, Ibaraki, Japan; 2 Tokunoshima Branch, Kagoshima Prefectural Institute for Agricultural Development (KIAD), Isen, Ōshima-gun, Kagoshima, Japan; 3 Vegetable Flower and Ornamental Plant Division, Saga Prefectural Agriculture Research Center, Kawasoe, Saga City, Saga, Japan; 4 Kumage Branch, KIAD, Nishinoomote, Nishinoomote City, Kagoshima, Japan; 5 Division of Plant Sciences, NIAS, Tsukuba, Ibaraki, Japan; Naval Research Laboratory, United States of America

## Abstract

Transcriptional gene silencing (TGS)–a phenomenon observed in endogenous genes/transgenes in eukaryotes–is a huge hindrance to transgenic technology and occurs mainly when the genes involved share sequence homology in their promoter regions. TGS depends on chromosomal position, suggesting the existence of genomic elements that suppress TGS. However, no systematic approach to identify such DNA elements has yet been reported. Here, we developed a successful novel screening strategy to identify such elements (anti-silencing regions–ASRs), based on their ability to protect a flanked transgene from TGS. A silenced transgenic tobacco plant in which a subsequently introduced transgene undergoes obligatory promoter-homology dependent TGS *in trans* allowed the ability of DNA elements to prevent TGS to be used as the screening criterion. We also identified ASRs in a genomic library from a different plant species (*Lotus japonicus*: a perennial legume); the ASRs include portions of Ty1/*copia* retrotransposon-like and pararetrovirus-like sequences; the retrotransposon-like sequences also showed interspecies anti-TGS activity in a TGS-induction system in *Arabidopsis*. Anti-TGS elements could provide effective tools to reduce TGS and ensure proper regulation of transgene expression. Furthermore, the screening strategy described here will also facilitate the efficient identification of new classes of anti-TGS elements.

## Introduction

Like that of endogenous genes, the expression of transgenes and activity of transposons/invasive nucleic acids can be influenced epigenetically [1], [2]. Transgene silencing can depend on the sequence homology of a transgene [1], [2], the degree of iteration of the transgene at the inserted location [3], [4], the chromosomal environment into which it is inserted [5], or the coincidence of some/all of these factors.

In plant research, transgene silencing has been categorized generally into two classes: transcriptional gene silencing (TGS) and post-transcriptional gene silencing (PTGS). TGS acts through prevention of transcription, and occurs mainly when the genes involved share sequence homology in their promoter regions [1], [2]; PTGS acts through sequence-specific degradation of transcripts and is dependent on homology within transcribed regions [1], [2]. TGS is widely recognized as a major hindrance to transgenic technology [6] because TGS arises spontaneously in transgenic plants and can be inherited in subsequent generations [3], [7], [8]. Therefore, the development of strategies that can prevent TGS is essential to the success of transgenic technology.

The fact that some transgenes undergo TGS while others do not [4]–[6] makes it conceivable that endogenous DNA sequences exist that actively determine the epigenetic TGS/non-TGS state of genomic regions. Transgene insert(s) showing TGS often force unlinked homologous promoters to be silenced *in trans* (*trans*-TGS) [9], [10]. Also, *trans*-TGS occurs on promoters in secondary-transfected (supertransformed) transgenes that are homologous to a pre-existing silenced promoter and inserted at a different locus/loci [11]–[13]. However, DNA sequences that uncouple transgenes from *trans*-TGS are unknown.

In this study, we sought to explore such genomic elements (hereafter referred to as anti-silencing regions–ASRs), which we hypothesized would actively protect a flanked transgene from TGS. First, we developed a novel screening strategy using a tobacco transgenic plant causing obligatory TGS when a transgene driven by the same promoter is supertransformed. We then used this strategy to isolate ASRs, based on their ability to suppress *trans*-TGS. We confirmed that one of the ASRs isolated in this study repressed TGS in all three assay systems tested, i.e., (1) a tobacco plant containing multiple transgene insertions and exhibiting obligatory TGS; (2) a tobacco plant carrying a homozygous 35S promoter-driven transgene in a single position, and showing TGS by increasing copy number of the promoter; and (3) an *Arabidopsis* line inducing obligatory silencing by transformation of an *FWA* genomic clone. Our findings also suggest that this ASR would exert anti-silencing activity in various plant species.

## Results

### Selection of an Obligatory *trans*-TGS Trigger Plant

**Figure 1:**
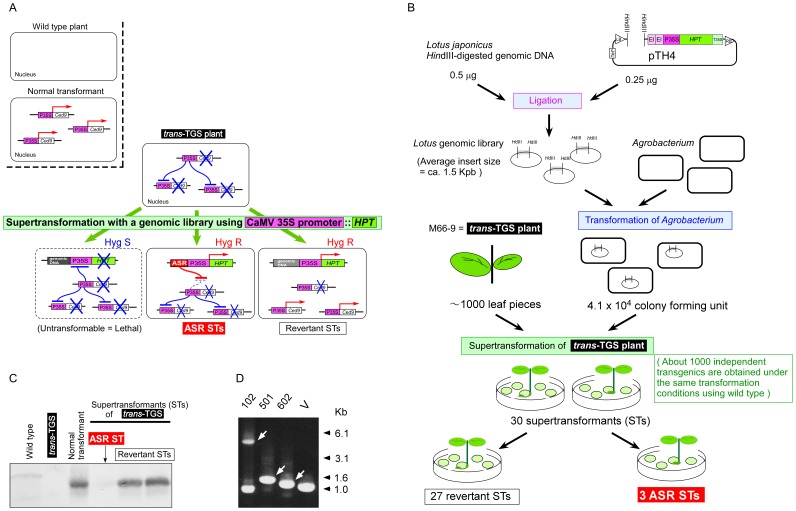
Identification of anti-silencing regions (ASRs) preventing transcriptional gene silencing (TGS): strategy to screen ASR candidates from a genomic DNA library. (**A**) Schematic diagram depicting strategies to select ASRs using a transgenic tobacco plant possessing *trans*-TGS activity. The Xs indicate that the transgenes are silenced. (**B**) Isolation of anti-silencing region (ASR) candidates. DNA elements that would have ASR activity were isolated from regenerated supertransformants of the *trans*-TGS plant (see also **A**). Under the same conditions of tobacco transformation, about 1000 independent supertransformed shoots are obtained when we use wild type ‘Samsun NN’. (**C**) Detection of restored expression from pre-existing transgene by Western blotting. For names of the lines, see **A**. (**D**) Isolation of ASR candidates. The arrows indicate ASR candidates amplified by PCR from three supertransformed *trans*-TGS plants using a library of ASR::*HPT* constructs (**A**, bottom right). A plant containing ASR102 showed the same size band as that generated by the empty vector (V), suggesting that this plant also has a genome insertion of the empty vector. V, vector used for the library construction.

We contrived a novel system to screen for ASRs based on promoter-homology causing obligatory TGS (Figure 1A). We first isolated a transgenic plant causing obligatory promoter-homology-dependent gene silencing of a “secondly”-introduced promoter. We have previously identified several TGS plants in transgenic tobacco transformed with an enhanced cauliflower mosaic virus (CaMV) 35S promoter::*bcl-x_L_*/*ced-9* construct [14] (hereafter P35S). These silencing plants had multiple copies of the transgene, transmitted the silencing status to the next generation, and exhibited reactivation of the transgene(s) upon treatment with 5-azacytidine–a potent inhibitor of DNA methylation–suggesting that the silencing mechanism was TGS. We then used these TGS plants as a resource to select an obligatory *trans*-TGS trigger plant (Figure S1), and identified M66-9 that could be used for ASR screening (Figures 1A).

### Isolation of ASR Candidates

Use of the obligatory *trans*-TGS plant, M66-9, allows us to isolate ASR candidates from DNA libraries constructed with selectable marker-harboring vectors. To construct a library as a resource for isolating ASR, we used genomic DNA fragments of *Lotus japonicus* and a binary vector, pTH4, that contains another enhanced P35S::*HPT* (hygromycin phosphotransferase gene) cassette (Figure 1B).

If P35S::*HPT* constructs containing genomic fragments with no ASR activity are supertransformed into explants of M66-9, the P35S in the construct would be silenced and no supertransformant would be obtained on hygromycin-containing medium (Figure 1A, bottom left). In contrast, supertransformants of M66-9 explants can be regenerated on the selection medium either if the supertransformed P35S::*HPT* construct is protected from *trans*-TGS by an adjacent ASR (Figure 1A, bottom middle) or if supertransformed cells lose their *trans*-TGS activity (revertants) (Figure 1A, bottom right). Because loss of *trans*-TGS activity will lead to reactivation of the pre-existing transgene (P35S::*ced-9*), revertants can be screened by detection of Ced-9 protein as an indicator (Figure 1C).

When about 1000 leaf pieces of M66-9 were inoculated with *Agrobacterium* containing the genomic library, a total of 30 independent supertransformed shoots were obtained (Figure 1B). To confirm that the pre-existing transgene (enhanced P35S::*ced*-9) still showed TGS, Ced-9 expression was examined in the 30 supertransformants by protein gel blot analysis. Ced-9 protein was detected in 27 out of the 30 plants, suggesting that these plants were epigenetic/genetic revertants from the silenced state (Figure 1C). We excluded these plants from further screening for ASRs because the original transgenes in these 27 plants had lost the *trans-*TGS activity (Figure 1A, bottom right, and 1C). The other three supertransformants showed no expression of the original transgene (Figure 1A, bottom middle, and 1C). These results indicated that the original transgenes in these three plants were silenced and that the plants had kept their *trans-*TGS activity, while the supertransformed P35S::*HPT* selectable marker genes in these plants were not silenced, suggesting that ASR activity was conferred by each genomic fragment inserted in the supertransformed construct (Figure 1A). Using a primer set designed within the vector sequence (Table S1) for PCR, we then isolated DNAs from three supertransformants as candidate ASRs (Figure 1D).

### ASR Candidates Include a Sequence Derived from Retrotransposons

The primary structures of the three ASR candidates were found to correspond to portions of the following sequences (Figure S2, Text S1); ASR102 (3 Kbp): an endogenous pararetrovirus-like sequence; ASR502 (0.3 Kbp): a “with-no-lysine” kinase-like sequence; ASR602 (171 bp): a Ty1/*copia* retrotransposon-like sequence. ASR602 itself has not been registered in any sequence database, while sequences similar to ASR602 (ASR602-containing retrotransposon-like sequences: ASLs) are highly species-specific and abundant in the *L. japonicus* genome (Figures S2C, D and S3).

### ASR Candidates have no Enhancer Activity

We next examined whether these ASR candidates have enhancer activity and can overcome TGS. Each of the three ASR candidates was inserted at the 5′ edge of a CaMV 35S minimal promoter::*GUS* (β-glucuronidase gene) cassette (Figure S4). None of the three genomic fragments inserted into this construct led to any significant increase in GUS expression (Table S2), indicating that these ASR candidates have no enhancer activity.

### Anti-silencing Activities of ASR Candidates in a P35S-dosage-dependent TGS System

**Figure 2:**
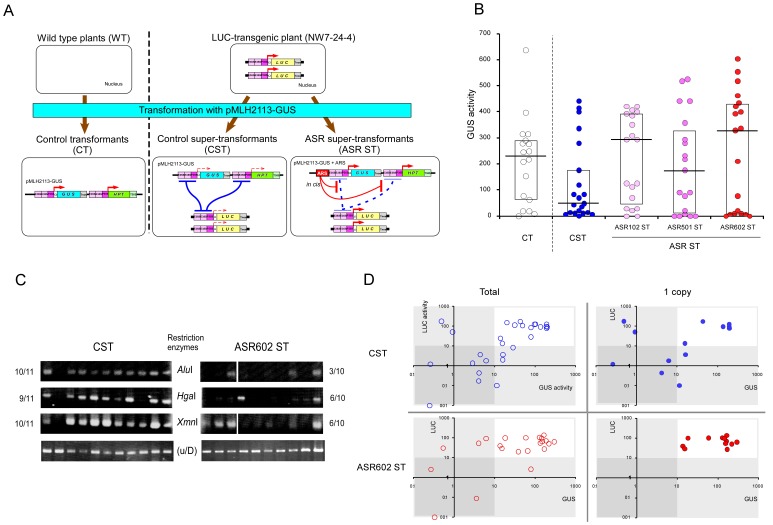
Anti-silencing activity of ASR candidates in preventing *trans*-TGS caused by interaction between CaMV 35S promoters. (**A**) Schematic representation of strategy to test if ASR candidates can prevent *trans*-TGS caused by increasing copy number of the same promoter. See text for details. The CT plants (T_0_ generation) were regenerated from primary transformed leaves of wild type plant “Samsun NN”. The ST and ASR ST plants (ST_0_ generation) were regenerated from primary supertransformed leaves of the *LUC*-transgenic tobacco plant, “NW7-24-4” [15]. (**B**) Prevention of *trans*-TGS by ASR candidates. The effects of ASRs on expression of the *GUS* gene driven by the 35S promoter (P35S) were examined. GUS activities of control plants (CT and CST) and supertransformants with one of the ASR candidates (ASR ST) are shown. Boxes show the range of the 25–75th quartiles of the data; the horizontal bars in the boxes present the median value of each group. GUS activity, 4-MU nmol mg protein^–1^ min^–1^. **4-MU**, 4-methylumbelliferone. (**C**) DNA methylation status of enhanced P35S promoters flanked (or not) with ASR602 in the GUS construct expressed in the P35S-dosage-dependent TGS system. The promoter regions of the GUS construct, pMLH2113-GUS, in control supertransformants (CST) and ASR supertransformants (ASR602 ST) were examined (Figures 2A and S4). Each lane represents the same supertransformant. The numbers to the right and left of the slashes represent total plants and plants harboring the methylated promoter, respectively. **u/D**, undigested DNAs. (**D**) Prevention of *trans*-TGS by ASR602. The activity of the pre-existing P35S::*LUC* insert was plotted against activity of the supertransformed P35S::*GUS* gene for CSTs and ASR602STs. LUC activity, photon µg protein^–1^ sec^–1^. Note that the scales are logarithmic. **Total**, all plants examined; **1 copy**, plants harboring a single copy of the P35S::*GUS* transgene.

To confirm the anti-silencing activities of the ASR candidates on different transgenes, we established a TGS-inducing system based on an increasing copy number of P35S regions (an inverse relationship between transgene copy number and expression level is often observed in homology-dependent TGS [3], [4], [9]). We had previously produced a transgenic tobacco plant (Figure 2A, NW7-24-4) harboring an enhanced P35S::*LUC* (luciferase gene) that exhibits a markedly high level of LUC expression [15]. Since this transgenic plant is a single-copy-inserted homozygote of the enhanced P35S::*LUC* construct, which itself carries two copies of the 35S enhancer region and one copy of the 35S promoter region, this plant has four copies of the 35S enhancer region and two copies of the 35S promoter region per diploid (Figure 2A). We supertransformed this enhanced P35S::*LUC* plant with pMLH2113-*GUS* (Figures 2A and S4). If this enhanced P35S::*GUS* construct is introduced into the enhanced P35S::*LUC* plant, the resulting supertransformants should have at least eight copies of the 35S enhancer region and four copies of the 35S promoter region (Figure 2A, CST, control supertransformant). When the enhanced P35S::*LUC* plant was supertransformed, about half of the resulting supertransformants showed an extremely low level of GUS expression (Figure 2B, CST). In contrast, when wild type tobacco was transformed with pMLH2113-*GUS*, most of the transformants exhibited relatively high GUS expression (Figure 2B, CT, control transformant), as reported with the same *GUS* cassette in a different vector [16]. The higher frequency of plants showing no/low GUS expression in the CST plants can be explained by TGS due to the increasing copy number of the 35S promoter in the supertransformants (Figure 2A and 2B, CST). Using this TGS-inducing system (hereafter, P35S-dosage-dependent TGS system), we next examined whether the three ASR candidates show genuine anti-silencing activities. ASR-containing pMLH2113-*GUS* constructs (Figure 2A, right) were introduced into the enhanced P35S::*LUC* plant. In supertransformants containing an ASR candidate (Figure 2B, ASR ST), the frequency of plants showing suppressed GUS activity was lower than that of supertransformed plants without ASR (Figure 2B, CST), suggesting that these ASR candidates exhibit anti-silencing activity also in this TGS-inducing system. ASR102 ST and ASR602 ST clearly showed higher medians of GUS activity than the control transformants/supertransformants (Figure 2B, CT and CST). The shortest sequence, ASR602 (171 bp), was chosen for further characterization.

### DNA Methylation Status of Enhanced P35S Promoters Flanked (or not) with ASR602 in the *GUS* Construct in a P35S-dosage-dependent TGS System

DNA methylation in promoter regions is often associated with TGS [17]. We analyzed the DNA methylation status of the 35S promoter region fused to the *GUS* gene in supertransformants with or without ASR602 (Figure 2A and 2B, CST and ASR602 ST) using a PCR-mediated methylation assay. The 35S promoter region fused with the *GUS* gene (yellow bar of pMLH2113-*GUS* in Figure S4) was digested using methylation-sensitive restriction endonucleases followed by PCR amplification (Table S1). If the promoter region is heavily methylated, the DNA in this region should be resistant to these enzymes, and PCR products from the region will be amplified. In most of the supertransformants without ASR602, amplified PCR products were detected after digestion with methylation-sensitive restriction enzymes, suggesting that the 35S promoter regions fused with *GUS* are heavily methylated in the supertransformants without ASR602 (Figure 2C, CST). On the contrary, amplified DNAs were barely detected in ASR602-containing supertransformants (Figure 2C, ASR602 ST). The results suggested that ASR602 might protect the adjacent promoter region from DNA methylation.

### Anti-*trans*-TGS Activity of ASR602 in the P35S-dosage-dependent TGS System

**Figure 3:**
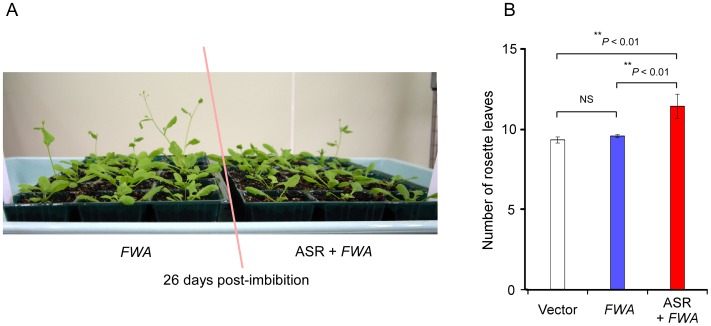
Silencing of the FWA transgene and its prevention by ASR602 in *Arabidopsis* (Col-0). (**A**) *FWA*-transformed plants (*FWA*) and plants transformed with an ASR602-fused *FWA* (ASR+*FWA*). (**B**) Flowering time in *FWA* transformants and control transformants (Vector: plants transformed with vector only). Late flowering increases the number of rosette leaves. Vector, n = 11; *FWA*, n = 117; ASR+*FWA*, n = 14; n, number of independent transformants. Mean values are given. Error bars denote ± s.e.m. **P<0.01; **NS**, not significant.

We next examined whether TGS is also induced at the pre-existing P35S::*LUC* locus in the same supertransformants (Figure 2D). Since supertransformation of the P35S::*GUS* should not alter the pre-existing P35S::*LUC* sequence, all the supertransformants should exhibit high levels of LUC expression. However, if the TGS induced by P35S::*GUS* supertransformation of the P35S::*LUC* plant is *trans*-TGS, expression of the pre-existing P35S::*LUC* would be affected (Figure 3A, CST).

We found a considerable number of supertransformed plants showing low levels of LUC expression (Figure 2D, left). In supertransformants without ASR602 (Figure 2D, top left, CST/Total), there was a significant positive correlation between P35S::*GUS* and P35S::*LUC* expression levels (Table S3); most of the supertransformants with low GUS expression also showed extremely low LUC expression, indicating that supertransformation with P35S::*GUS* induced *trans-*TGS of P35S::*LUC* (Figure 2D, top left, CST/Total). We then examined whether TGS of P35S::*LUC* is caused when a P35S::*GUS* flanked with ASR602 is supertransformed. In supertransformants containing ASR602 (ASR602 ST), a very small number of plants showed low expression of P35S::*GUS* and/or P35S::*LUC* transgenes (Figure 2D, bottom left, ASR602 ST/Total), suggesting that supertransformation with the P35S::*GUS* containing ASR602 suppresses *trans*-TGS of the P35S::*LUC* as a result of preventing TGS of the P35S::*GUS*.

Plants harboring multiple copies of a transgene tend to have complicated transgenic loci [18] and/or undergo PTGS [2], [15]. We measured the copy number of P35S::*GUS* to select supertransformants containing only a single copy of P35S::*GUS* (Figure 2D, right, 1 copy) from all the plants examined (Figure 2D, left, Total). To determine the copy number of P35S::*GUS* in the supertransformants, gene-dosage ratios of *GUS*/*LUC* were measured with real-time PCR using genomic DNAs isolated from the supertransformants (Table S1). We re-plotted GUS and LUC expression data of the selected supertransformants (Figure 2D, right, 1 copy). In supertransformants without ASR602 (Figure 2D, top right, CST/1 copy), about half the plants (7/13) showed low expression levels of the pre-existing P35S::*LUC* and/or the supertransformed P35S::*GUS*. In supertransformants with ASR602 (Figure 2D, bottom right, ASR602 ST/1 copy), no plants (0/12) expressed low levels of the P35S::*LUC* and/or P35S::*GUS* transgenes. These results show that ASR602 effectively repressed the TGS/*trans*-TGS induced by a single insertion of the supertransformed gene.

### Anti-silencing Activity of ASR602 on TGS of the *FWA* Gene Promoter in *Arabidopsis thaliana*

To test whether ASR602 shows anti-silencing activity in different plant species and with different promoters, we transformed wild type *Arabidopsis thaliana*, ecotype Colombia (Col-0) using a genomic clone of the *Arabidopsis* flowering gene *FWA* with/without ASR602 (Figure S4). The endogenous gene is heritably silenced, and direct repeats at the 5′-end of its transcribed region are methylated in most tissues; a hypomethylated *fwa* mutant shows ectopic overexpression of *FWA*, resulting in late flowering [19]. Wild-type plants transformed with an *FWA* genomic clone, including the direct repeats, silence the *FWA* transgene efficiently and do not alter flowering time [19], [20].

If ASR602 has an anti-silencing activity, wild-type plants transformed with an ASR602-fused *FWA* gene should exhibit late flowering. Plants transformed with an *FWA* genomic clone without ASR602 (Figure 3, *FWA*) showed the same flowering time as vector control transformants (Figure 3B, Vector) and wt Col-0 plants, indicating *FWA* transgene silencing. Plants transformed with an *FWA* genomic clone flanked with ASR602 (Figure 3, ASR+*FWA*) showed significantly later flowering compared with both transformants of *FWA* without ASR (Figure 3, *FWA*) and vector control transformants (Figure 3B, Vector), indicating that ASR602 inhibits silencing of the *FWA* transgene. It would also appear that ASR602 might exert anti-TGS activity in the next generation (Figure S5). Taken together, these results suggest that ASR602 exhibits cross-species TGS suppression activity.

## Discussion

We have developed a novel screening system based on anti-silencing ability in order to isolate regulatory DNAs reducing TGS. ASR602 is the first DNA element with interspecies-wide anti-silencing activity to be identified using this assay system. By employing three different *trans*-TGS trigger systems using different constructs and promoters, we showed that transgenes linked to ASR602 were able to circumvent *trans*-TGS. We identified ASR602, based on its activity to prevent a supertransformed P35S-driven construct from *trans*-TGS, as screening criterion (Figures 1A) and confirmed the anti-TGS activity by the different TGS-inducing system using “NW7-24-4”, the highly-expressed P35S::*LUC* tobacco plant (Figure 2A). It is not until the *LUC*-transgenic plant is supertransformed with a P35S-driven construct (e.g. the P35S::*GUS* construct, Figure 2A) that TGS occurs in the *LUC* plant. In this respect, the TGS-inducing system using the *LUC* plant is different from the ASR screening system using the *trans*-TGS plant, “M66-9”, in which TGS already occurs at the pre-existing P35S::*Ced-9* transgene loci (Figures 1 and S1B). In the TGS-inducing system (Figure 2A), it would appear that the supertransformed P35S::*GUS* construct become a TGS trigger locus(loci), causing not only TGS on the supertransformed construct itself but also *trans*-TGS on the pre-existing *LUC* locus after supertransformation, although the mechanisms that make the supertransformed construct a TGS trigger locus(loci) remain unknown. We speculate that ASR602 would first hamper TGS initiation on the supertransformed ASR602::P35S::*GUS* construct, simultaneously preventing the supertransformed construct from becoming a TGS trigger locus(loci), and consequently TGS would not occur on the pre-existing *LUC* locus.

ASR602, a species-specific sequence obtained from a genomic DNA library of *L. japonicus*, had no sequence characteristics similar to known scaffold/matrix attachment regions (S/MARs) using three prediction tools (Text S1). Because we have not assessed experimentally whether ASRs can influence chromatin structure, our results at present do not rule out the possibility that ASRs have S/MAR-like or insulator-like activities. S/MARs and barrier insulators have been identified as boundary elements in the genomes of several species, and can stabilize expression when used to flank transgenes. Such elements uncouple (trans)genes from position effects such as spreading of a heterochromatic region into adjacent euchromatic regions [21], [22]. On the other hand, when fused to a transgene, ASR602 suppressed TGS caused by a silencer locus (loci) located on a separate chromosome(s)/region(s). This suggests that ASR602 acts as a “cue” to determine the active status of chromatin as controlled by the epigenetic regulation systems of the genome. Regulatory factors that directly bind to, or indirectly associate with, ASR602-like sequences could recruit activation factors and/or ward off repression factors [23]. The epigenetic status of the genome would be controlled by both boundary elements, which compartmentalize genomic regions to be regulated epigenetically, and “cue elements”, like ASR602, that keep genomic regions active epigenetically (or prevent such regions from being inactive). Genomic elements with anti-silencing activity, like ASR602, might be abundant in the genomes of eukaryotic organisms. Further larger-scale screening could lead to the isolation of many other anti-silencing elements with various characteristics/structures.

An intriguing possibility is that ASR602–the first reported “cue element” to determine the epigenetic active status of chromatin–has functions similar to boundary elements. Although two DNA sequences with barrier insulator activity have been identified previously from *Drosophila* Ty3/*gypsy* retrotransposons [24], [25], their anti-*trans*-TGS activities have not been determined. Although functions of ASR602, which originated from a Ty1/*copia* retrotransposon, as a boundary element have not been determined experimentally, supertransformed P35S::*LUC* tobacco plants harboring single copy of the ASR602::P35S::*GUS* construct (Figure 2D, bottom right, ASR602 ST/1 copy) included no supertransformants showing TGS, suggesting that no position effect on the *GUS* transgene occurs in these supertransformants (ASR602 ST/1 copy), and that the function of boundary elements in preventing position effects and those of “cue elements” like ASR602 could overlap rather than be mutually exclusive. The screening strategy described here will facilitate the efficient identification of novel anti-TGS elements.

The mechanisms underlying the anti-silencing activity of the ASRs isolated in this study remains to be determined in further investigations, and will certainly be intriguing. Protection from DNA methylation of promoters linked with ASRs might be crucial to the underlying mechanisms (Figure 2C).

We speculate that some retrotransposons/retroviruses have evolved specific sequences/activities to break down host defense systems. PTGS forms a major part of the defense system of plants against RNA viruses, and many plant RNA viruses can combat defense systems using viral PTGS suppressors [1], [2]. Some plant DNA viruses have also been suggested to have the ability to counteract the TGS responses of their hosts [26]. Since TGS forms part of the genome surveillance system that prevents multiplication of parasitic DNAs/transposons [1], [9], [27], it is conceivable that TGS poses a threat to retrotransposons in host genomes, and that, in response, some retrotransposons have evolved counter-defense activities.

The concepts developed for this screening strategy could be applied to the isolation of ASRs from a wide range of organisms. ASRs isolated by this method could lead to significant breakthroughs in transgenic technology, and analyses of ASRs will open up new research topics in the field of epigenetic mechanisms of genome regulation.

## Materials and Methods

### Plant Materials

Plants and cultured cells of wild type (*Nicotiana tabacum* cv. Samsun NN) and transgenic tobacco were grown as described [15]. To select a plant showing *trans*-TGS activity, plants from transgenic tobacco lines M65 and M66 were used [14]. The *LUC*-transgenic tobacco plant, “NW7-24-4”, is described elsewhere [15]. *Agrobacterium*-mediated transformation of tobacco and *Arabidopsis thaliana* was carried out as described elsewhere [15], [28]. Flowering time of *Arabidopsis* ecotype Col-0 and the transformants was measured by counting the number of rosette leaves of plants grown under long-day conditions (16 hr light/8hr dark). Flowering time data shown in Figure 3 were compared with Dunn’s multiple comparison procedure [29]. The other statistical methods were carried out according to a standard textbook of biostatistics [30], except as otherwise noted.

### Biochemical Analysis

Genomic DNA extraction from tobacco and *Arabidopsis* was carried out as described [15]. Genomic DNA of *Lotus japonicus* (accession Miyakojima MG-20) was provided by Y. Umehara (NIAS). A polyclonal antibody to Ced-9 was used for immunoblotting [31].

### Plasmid Construction

pTH1, which contains a P35S::*HPT* (hygromycin phosphotransferase gene) as a selectable marker, has the same features as the pTH2 vector [32] except that the direction of the *HPT* expression cassette is reversed. pMLH7133-GUS–a derivative of pMLH7133-*mwti1b* (which contains *HPT*) [33]–was constructed by replacing the *mwti1b* gene with the *GUS* reporter gene of the promoter-*GUS* cassette in pE7133-GUS [16]. To generate pTH4, the *HPT* expression cassette of pMLH7133-*mwti1b* (*Sma*I-*Sma*I region) [33] was inserted into the *Xho*I site (flush-ended with Klenow fragment) of pTRA415(R)-delNPT [34]. To create the *Lotus* genomic library, genomic DNA was digested with *Hin*dIII and ligated into pTH4 (Figure S4).

To generate pP35Sm-GUS, the CaMV promoter region of pBI121 [35] was replaced by the –46 CaMV 35S minimal promoter (P35Sm) [36]. ASR candidates were inserted into the *Hin*dIII site located immediately upstream of P35Sm in this construct and tested for enhancer activity (Figure S4). pMLH2113-GUS was derived from pMLH7133-GUS by replacing the promoter-*GUS* cassette with the promoter-*GUS* cassette of pE2113-GUS (*Hin*dIII–*Bam*HI fragment) [16].

To generate a ‘control’ construct for *FWA* transformation (pBI-FWA, Figure S4), a 5.5-Kb fragment of the *FWA* gene, which corresponds to positions 13037026 to 13042519 in GenBank accession NC_003075, was PCR-amplified (with primer set ‘UP FWA51’ bearing an introduced *Hin*dIII site, and ‘FWA32’ bearing an endogenous *Eco*RI site; Table S1) from genomic DNA of *Arabidopsis* (ecotype Col-0). The promoter-*GUS* reporter cassette of pBI121 (*Hin*dIII–*Eco*RI fragment) [35] was replaced with the PCR product digested with *Hin*dIII and *Eco*RI. To generate the ASR602-containing construct for *FWA* transformation, a 172-bp region between the introduced *Hin*dIII site and an endogenous *Xba*I site of pBI-FWA was replaced with an ASR602 fragment with an introduced *Xba*I site to which the 3′ end of the original ASR602 (171 bp *Hin*dIII fragment) was converted by PCR.

### Identification of Eight ASR602-containing Retrotransposon-like Sequences (ASLs)

First, we used the DNA sequence of ASR602 (171 bp) as a query sequence for BLASTN analysis on the GenBank web site (http://blast.ncbi.nlm.nih.gov/Blast.cgi) with default conditions, except using ‘Database’ = ’Others (nr etc.)’, resulting in a list of accession numbers including ASLs based on E-values. Second, we used sequence data of the ‘top 20’ accession numbers to find all ASLs with the 20th E-value or with an E-value less than the 20th’s. Third, in such ASLs (E-values≤the 20th’s E-value), we looked for ASLs that were sandwiched between a pair of LTRs with target site duplication. Finally, we identified the eight ASLs shown in Figure S2C. Eight ASLs (ASL1-ASL8) were found in sequence data of following accession numbers in order, respectively: AP010575, AP006141, AP009783, AP009654, AP009679, AP010505, AP006114 and AP004952.

### GUS and LUC Assays

Reporter gene assays were performed as described [36]. An aliquot of cell extract was incubated in buffer containing 4-methylumbelliferyl-β-d-glucuronide at 37°C for 30 min. To measure GUS activity, the quantity of 4-methylumbelliferone formed was determined using a fluorescence spectrophotometer (F-2500; Hitachi High-Tech Co., Ltd.). A 20-µl aliquot of the supernatant was incubated in 50 µl of PicaGene™ (a solution for LUC assay; Toyo B-Net Co., Ltd.). LUC activity was determined for 10 sec using a Microtiter Plate Luminometer MLX™ (Dynex).

### Qualitative PCR Assay to Detect DNA Methylation Using Methylation-sensitive Enzymes

The basic concept of this assay is described elsewhere [37]. Tobacco plants analyzed for methylation status were chosen randomly from among supertransformed tobacco plants with/without ASR602 (Figure 2B and 2D, CST and ASR602 ST). Genomic DNAs were isolated individually and digested with a methylation-sensitive restriction enzyme: *Alu*I, *Hga*I or *Xmn*I. Each DNA sample (100 ng) was digested with 0.5 units of each restriction enzyme for 2 hr according to the manufacturer’s instructions, followed by ethanol precipitation. For PCR amplification, 75 ng of each digested DNA was used as template with AmpliTaq Gold DNA Polymerase (Applied Biosystems) in 10 µl reactions, according to the manufacturer’s recommendations. The cycling conditions were as follows: 95°C for 9 min; 40 cycles of 30s annealing at 55°C, 1 min elongation at 72°C, and 30 s denaturation at 95°C; 72°C for 5 min. The 35S promoter region fused with the *GUS* gene (yellow bar in pMLH2113-*GUS* in Figure S4) was PCR amplified with the primer set ‘pBI-Hind3-51’ and ‘GUSI3’, which correspond to a sequence 5′ upstream of the promoter region and a sequence at the 5′ end region of *GUS*, respectively (Table S1). Half of the volume of each PCR product was subjected to gel-electrophoresis.

### Quantitative PCR Assay to Determine Transgene Copy Number in Supertransformants

pMLH2113-GUS construct copy number was quantified by measuring gene-dosage ratios of *GUS*/*LUC* in each supertransformant, using real time PCR as described [38]. DNAs were isolated individually from all of the transformed and supertransformed plants whose GUS and LUC activities were measured. The *GUS* and *LUC* genes were PCR amplified with two primer pairs (*GUS*: ‘GUSI51’ and ‘Tnos Yomeru’; *LUC*: ‘LUCI51’ and ‘Tnos Yomeru’), respectively (Table S1).

### Accession Number

The sequence of ASR602 reported in this paper has been deposited with the DDBJ database under accession number AB632368.

## Supporting Information

Figure S1 **Identification of an obligatory** ***trans*** **-TGS trigger plant.** (**A–C**) Identification of an obligatory *trans*-TGS trigger plant, M66-9, by supertransformation with a P35S-driven *HPT* gene. To select plants showing *trans*-TGS activity, plants from transgenic tobacco lines M65 and M66 were used [14] (**A**). M65 and M66 lines harbor the open reading frames *bcl-xL*, and *ced-9*, respectively [31], inserted into the expression vector pBE2113 [16]. pBE2113 has two copies of the enhancer region (El; –419 to –90) of the CaMV 35S promoter (P35S), and one copy of the core promoter (–90 to –1) of P35S followed by a gene of interest to be expressed. We have previously identified several TGS plants in M65 and M66 [14]. To select a plant showing obligatory *trans-*TGS activity, we supertransformed explants of these TGS plants with the P35S::*HPT* construct pTH1 (Figure S4) (**A** and **B**). If any of these TGS plants had a potent *trans-*TGS activity, pTH1 would be silenced and supertransformation would lead to neither callus induction nor shoot regeneration from the explants on selective medium. Indeed, such a line was found: no callus or shoots were obtained from TGS plant M66-9 upon supertransformation with pTH1 (**C**), suggesting that M66-9 has *trans-*TGS activity. The red circles in **C** indicate regenerating shoots. (**D**) M66-9 becomes infected with *Agrobacterium* and confers obligatory *trans*-TGS on another 35S promoter-driven construct. We supertransformed M66-9 with another P35S-driven construct, pMLH7133-GUS, which contains the *HPT* and *GUS* genes, each driven by an enhanced P35S (Figure S4). At two days after infection with *Agrobacterium* containing this construct, M66-9 explants show GUS staining spots. However, the explants no longer showed any GUS spots at seven days after infection, nor did they regenerate any supertransformed shoots, indicating that the *GUS* construct is introduced into M66-9 explants but subjected to *trans*-TGS thereafter. M66-9 was thus identified as an obligatory *trans*-TGS triggering plant.
(TIF)

Figure S2 **Primary structure of three ASR candidates (see also Text S1).** (**A**) Schematic structures of ASR102 (not to scale). Stippled regions illustrate ranges showing amino acid similarity between the *Lotus* sequence and the virus. Percentages of “AA identities” and “AA positives” represent deduced similarities of amino acid sequences obtained using TBLASTN of GenBank (query: NP_127504, subject: ASR102 sequence) with default parameters. **MP**, viral movement protein; **ZF**, zinc finger domain; **PRO**, protease; **RT**, reverse transcriptase; **RH**, RNase H. (**B**) Schematic structures of ASR501 (not to scale). Percentages of “nt identities” represent nucleotide similarities obtained using BLASTN with default parameters. (**C**) ASR602-containing retrotransposon-like sequences (ASLs) and two Ty1/*copia* retrotransposons, Tnt1 (tobacco, X13777) and *copia* (*Drosophila*, X02599). In ASL1, a region corresponding to the nucleic acid-binding protein (NAB), protease genes and a part of the integrase (INT) gene is deleted. Stippled boxes show insertion; different stippled patterns represent different sequences. **LTR**, long terminal repeats; **PBS**, primer binding site. **NAB**, nucleic acid-binding protein; **INT**, integrase; **PPT**, polypurine tract. (**D**) DNA sequence alignment of ASR602 (171 bp) and ASR602-like sequences in the eight ASLs. (**D**) Amino acid similarity among the reverse transcriptase domains of Tnt1 (P10978), *copia* (P04146) and three ASLs (ASL1, 2, and 8). Amino acid positions of Tnt1 and *copia* (in parentheses) refer to those used in the accession numbers.
(TIF)

Figure S3 **Chromosomal distribution of** ***Lotus*** **DNA sequences similar to ASR602.** (**A**) Ratios of accession numbers containing ASR602-like sequences to accession numbers at http://www.kazusa.or.jp/lotus/clonelist.html. “Contigs harboring ASR602-like sequences” (in red) consist of accession numbers including one or more ASR602-like sequences. “Contigs without ASR602-like sequences” (in blue) consist of accession numbers that do not contain ASR602-like sequence. **n**, number of non-redundant accession numbers assigned to each chromosome or categorized as unmapped contigs. (**B**) Sequences similar to ASR602 were mapped on a genetic map of *L. japonicus* (http://www.kazusa.or.jp/lotus/clonelist.html) at 1 centimorgan (cM) intervals. To find similar sequences, the “bl2seq” search (a specialized BLAST to align two sequences on http://blast.ncbi.nlm.nih.gov/Blast.cgi) was used with default parameters with the ASR602 sequence as the query and with each non-redundant accession number on http://www.kazusa.or.jp/lotus/clonelist.html as the subject. The identified ASR602-like sequences were plotted at the map position (cM) (http://www.kazusa.or.jp/lotus/clonelist.html) of the accession number used as the subject. Filled circles, E-value <10^–20^; Open circles, E-value >10^–20^. Horizontal lines and vertical bars under the x-axes indicates regions containing centromeres and the genetic markers used as *in situ* hybridization probes [a marker on chromosome 1 (the dashed part) is not mapped on the genetic map], respectively [39].
(TIF)

Figure S4 **Binary vector constructs used for this study (see also Materials and Methods).** pTH1 was used to supertransform TGS plants for selection of plants showing *trans*-TGS activity. This vector contains the CaMV 35S enhancer region (E35S) and promoter region (P35S) of pBI121, followed by *HPT*. **RB** and **LB**, right and left borders of T-DNA of *Agrobacterium tumefaciens* Ti plasmid, respectively. **E35S**, 5′-upstream sequence of CaMV 35S promoter (–940 to –90). **P35S**, 5′-upstream sequence of CaMV 35S promoter (–90 to –1). ***HPT***, hygromycin phosphotransferase gene (a selectable marker). **Tnos**, polyadenylation signal of the nopaline synthase gene (*nos*) in the Ti plasmid. ***Tet^R^***, a tetracycline-resistance marker gene. pMLH7133-GUS was used as a second CaMV 35S promoter-driven construct to confirm *trans*-TGS activity of the *trans*-TGS plant M66-9. The GUS expression cassette in this construct contains seven copies of the CaMV 35S enhancer (E7) and the P35S. **Pnos::** ***NPTII***, *nos* promoter-driven neomycin phosphotransferase gene (a selectable marker) that confers resistance to kanamycin. **E7**, 5′-upstream sequence of CaMV 35S promoter (–940 to –290) and (–290 to –90) x 7. **Ω**, 5′-untranslated sequence of tobacco mosaic virus. **In**, first intron of a phaseolin gene. ***GUS***, β-glucuronidase gene (a reporter gene). **T35S**, polyadenylation signal of the CaMV 35S transcript. pTH4 was used to construct the genomic library of *L. japonicus* for ASR screening. This vector contains two copies of the CaMV 35S enhancer (El) and the P35S. **El**, 5′-upstream sequence of CaMV 35S promoter (–419 to –90). pP35Sm-GUS was used to examine whether an ASR candidate has enhancer activity. This construct harbors the –46 CaMV 35S minimal promoter region. pMLH2113-GUS was used to supertransform the *LUC* tobacco plant (Figure 3A, NW7-24-4). This construct carries two copies of El and the P35S. The yellow bar depicts the region where the methylation status was analyzed by methylation-sensitive restriction enzyme-coupled PCR assay (Figure 3C). Two arrows flanking the yellow bar (pBI-Hind3-51 and GUSI3) indicate the primer set used for the PCR assay (Table S1). pBI-FWA and an ASR-containing pBI-FWA were used to examine if ASRs prevent silencing of the *FWA* transgene in *Arabidopsis*. A 5′ end portion of the *FWA* region cloned in pBI-FWA was replaced with the ASR602 sequence, resulting in ASR602-containing pBI-FWA. Both FWA constructs contain the SINE-related tandem repeats of *FWA* gene (TR), which are sufficient to trigger *de novo* DNA methylation [40], [41].
(TIF)

Figure S5 **Flowering time and** ***FWA*** **expression of T_1_ generation derived from the T_0_ transformants, “** ***FWA*** **” and “ASR+** ***FWA*** **”, shown in** **Figure 3** **.** (**A**) Flowering time. Each triangle/dot depicts a T_1_ plant individual. T_1_ seeds were collected in bulk from each T_0_ generation group. (**B**) Quantitative real-time polymerase chain reaction analysis of *FWA* RNA in leaf of T_1_ plant. Methods of the PCR analysis was described elsewhere [42]. Prime sets used were shown in Table S1. wt (Col-0), n = 3; *FWA* (T_1_), n = 12; ASR+*FWA* (T_1_), n = 11; n, number of plant individuals. Mean values are given. Error bars denote ± s.e.m.
(TIF)

Table S1 **Oligonucleotide primers used in this study.**
(DOC)

Table S2 **Anti-silencing regions (ASR) do not have enhancer activity.**
(DOC)

Table S3 **Pearson’s correlation between the P35S::GUS and the P35S::LUC expression levels in** **Figure 2D** **.**
(DOC)

Text S1 **Primary structure of three ASR candidates.** (References for supporting information are compiled as “**Supporting Information References**” on the last page of the.doc file Text S1.
(DOC)
