# Supplementary figures and images for: DNA Elements Reducing Transcriptional Gene Silencing Revealed by a Novel Screening Strategy
Source: PLoS One. 2013 Jan 30;8(1):e54670. doi: 10.1371/journal.pone.0054670 (PMC3559876; doi:10.1371/journal.pone.0054670)

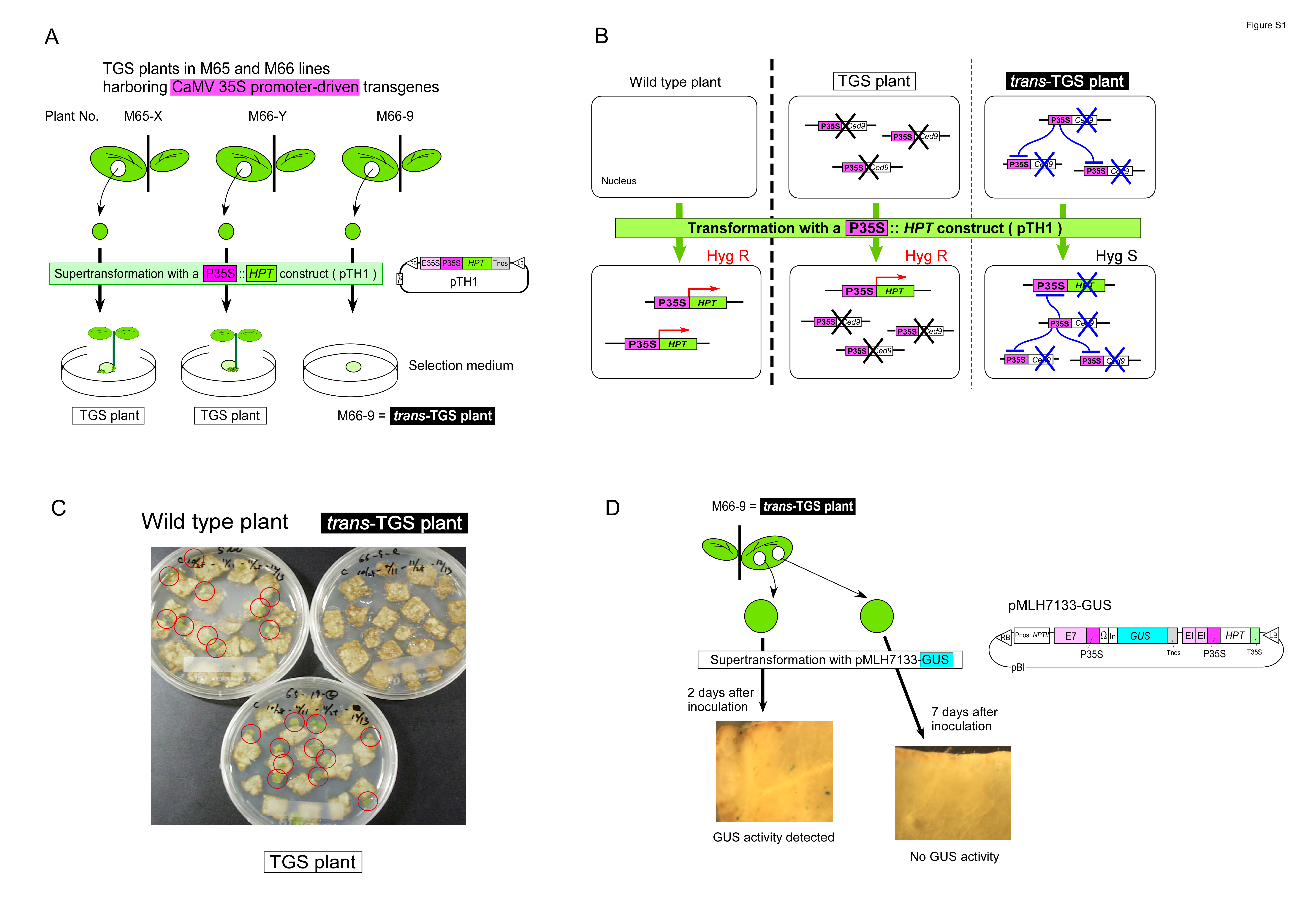

Supplement: Figure S1 — Identification of an obligatory trans -TGS trigger plant. (A–C) Identification of an obligatory trans-TGS trigger plant, M66-9, by supertransformation with a P35S-driven HPT gene. To select plants showing trans-TGS activity, plants from transgenic tobacco lines M65 and M66 were used [14] (A). M65 and M66 lines harbor the open reading frames bcl-xL, and ced-9, respectively [31], inserted into the expression vector pBE2113 [16]. pBE2113 has two copies of the enhancer region (El; –419 to –90) of the CaMV 35S promoter (P35S), and one copy of the core promoter (–90 to –1) of P35S followed by a gene of interest to be expressed. We have previously identified several TGS plants in M65 and M66 [14]. To select a plant showing obligatory trans-TGS activity, we supertransformed explants of these TGS plants with the P35S::HPT construct pTH1 (Figure S4) (A and B). If any of these TGS plants had a potent trans-TGS activity, pTH1 would be silenced and supertransformation would lead to neither callus induction nor shoot regeneration from the explants on selective medium. Indeed, such a line was found: no callus or shoots were obtained from TGS plant M66-9 upon supertransformation with pTH1 (C), suggesting that M66-9 has trans-TGS activity. The red circles in C indicate regenerating shoots. (D) M66-9 becomes infected with Agrobacterium and confers obligatory trans-TGS on another 35S promoter-driven construct. We supertransformed M66-9 with another P35S-driven construct, pMLH7133-GUS, which contains the HPT and GUS genes, each driven by an enhanced P35S (Figure S4). At two days after infection with Agrobacterium containing this construct, M66-9 explants show GUS staining spots. However, the explants no longer showed any GUS spots at seven days after infection, nor did they regenerate any supertransformed shoots, indicating that the GUS construct is introduced into M66-9 explants but subjected to trans-TGS thereafter. M66-9 was thus identified as an obligatory trans-TGS triggering plan [file pone.0054670.s001.tif]

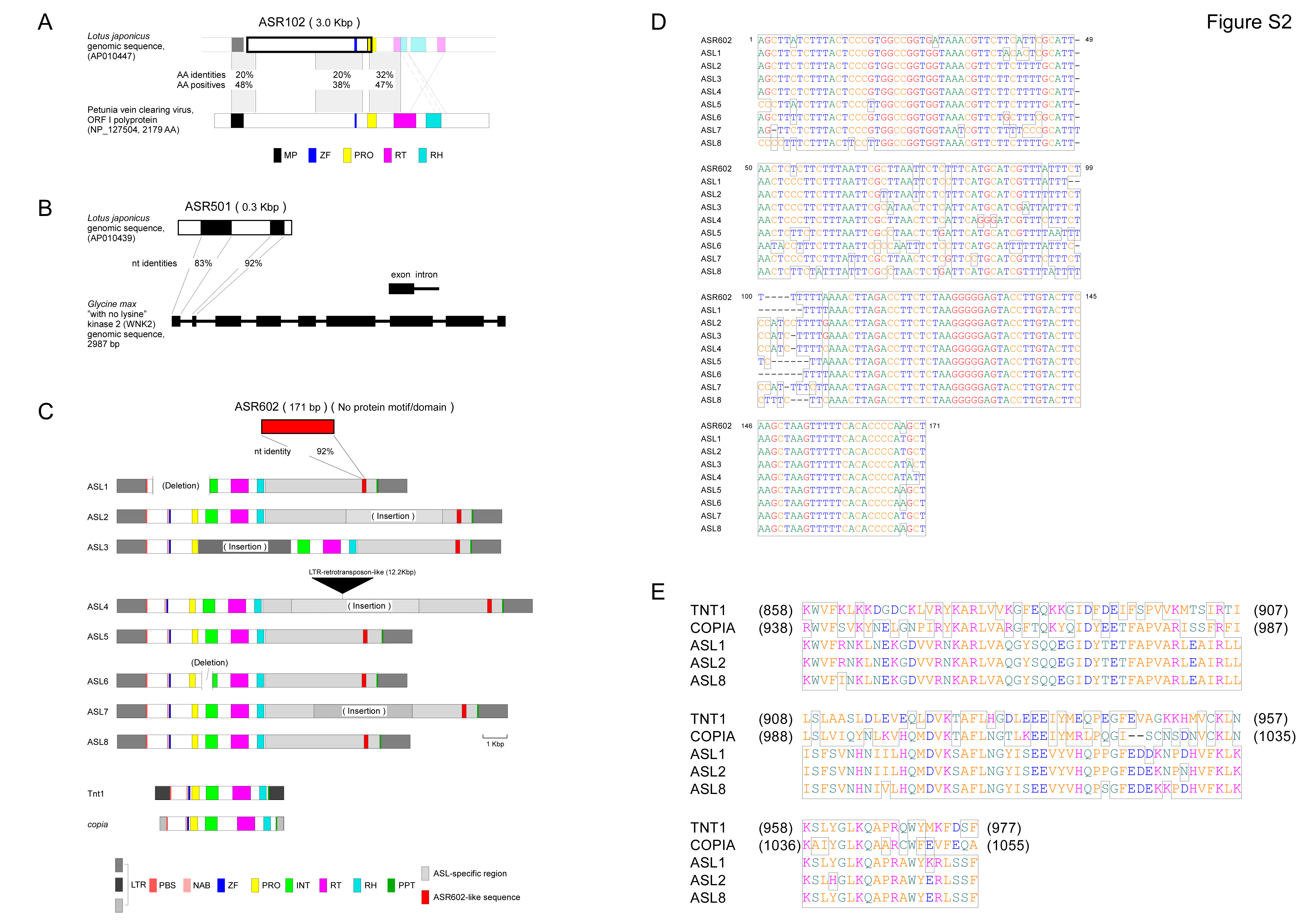

Supplement: Figure S2 — Primary structure of three ASR candidates (see also Text S1). (A) Schematic structures of ASR102 (not to scale). Stippled regions illustrate ranges showing amino acid similarity between the Lotus sequence and the virus. Percentages of “AA identities” and “AA positives” represent deduced similarities of amino acid sequences obtained using TBLASTN of GenBank (query: NP_127504, subject: ASR102 sequence) with default parameters. MP, viral movement protein; ZF, zinc finger domain; PRO, protease; RT, reverse transcriptase; RH, RNase H. (B) Schematic structures of ASR501 (not to scale). Percentages of “nt identities” represent nucleotide similarities obtained using BLASTN with default parameters. (C) ASR602-containing retrotransposon-like sequences (ASLs) and two Ty1/copia retrotransposons, Tnt1 (tobacco, X13777) and copia (Drosophila, X02599). In ASL1, a region corresponding to the nucleic acid-binding protein (NAB), protease genes and a part of the integrase (INT) gene is deleted. Stippled boxes show insertion; different stippled patterns represent different sequences. LTR, long terminal repeats; PBS, primer binding site. NAB, nucleic acid-binding protein; INT, integrase; PPT, polypurine tract. (D) DNA sequence alignment of ASR602 (171 bp) and ASR602-like sequences in the eight ASLs. (D) Amino acid similarity among the reverse transcriptase domains of Tnt1 (P10978), copia (P04146) and three ASLs (ASL1, 2, and 8). Amino acid positions of Tnt1 and copia (in parentheses) refer to those used in the accession numbers. (TIF) [file pone.0054670.s002.tif]

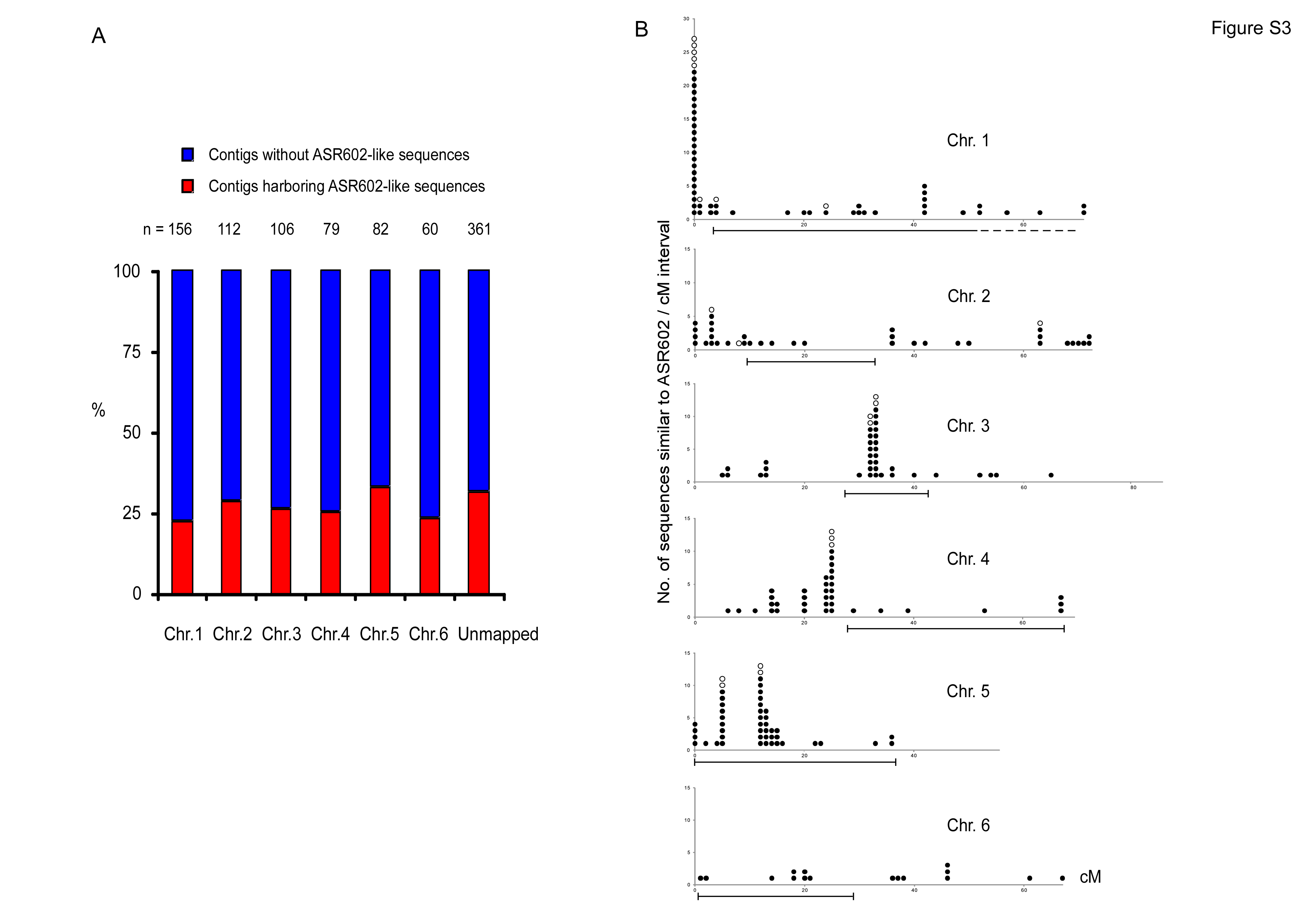

Supplement: Figure S3 — Chromosomal distribution of Lotus DNA sequences similar to ASR602. (A) Ratios of accession numbers containing ASR602-like sequences to accession numbers at http://www.kazusa.or.jp/lotus/clonelist.html. “Contigs harboring ASR602-like sequences” (in red) consist of accession numbers including one or more ASR602-like sequences. “Contigs without ASR602-like sequences” (in blue) consist of accession numbers that do not contain ASR602-like sequence. n, number of non-redundant accession numbers assigned to each chromosome or categorized as unmapped contigs. (B) Sequences similar to ASR602 were mapped on a genetic map of L. japonicus (http://www.kazusa.or.jp/lotus/clonelist.html) at 1 centimorgan (cM) intervals. To find similar sequences, the “bl2seq” search (a specialized BLAST to align two sequences on http://blast.ncbi.nlm.nih.gov/Blast.cgi) was used with default parameters with the ASR602 sequence as the query and with each non-redundant accession number on http://www.kazusa.or.jp/lotus/clonelist.html as the subject. The identified ASR602-like sequences were plotted at the map position (cM) (http://www.kazusa.or.jp/lotus/clonelist.html) of the accession number used as the subject. Filled circles, E-value <10–20; Open circles, E-value >10–20. Horizontal lines and vertical bars under the x-axes indicates regions containing centromeres and the genetic markers used as in situ hybridization probes [a marker on chromosome 1 (the dashed part) is not mapped on the genetic map], respectively [39]. (TIF) [file pone.0054670.s003.tif]

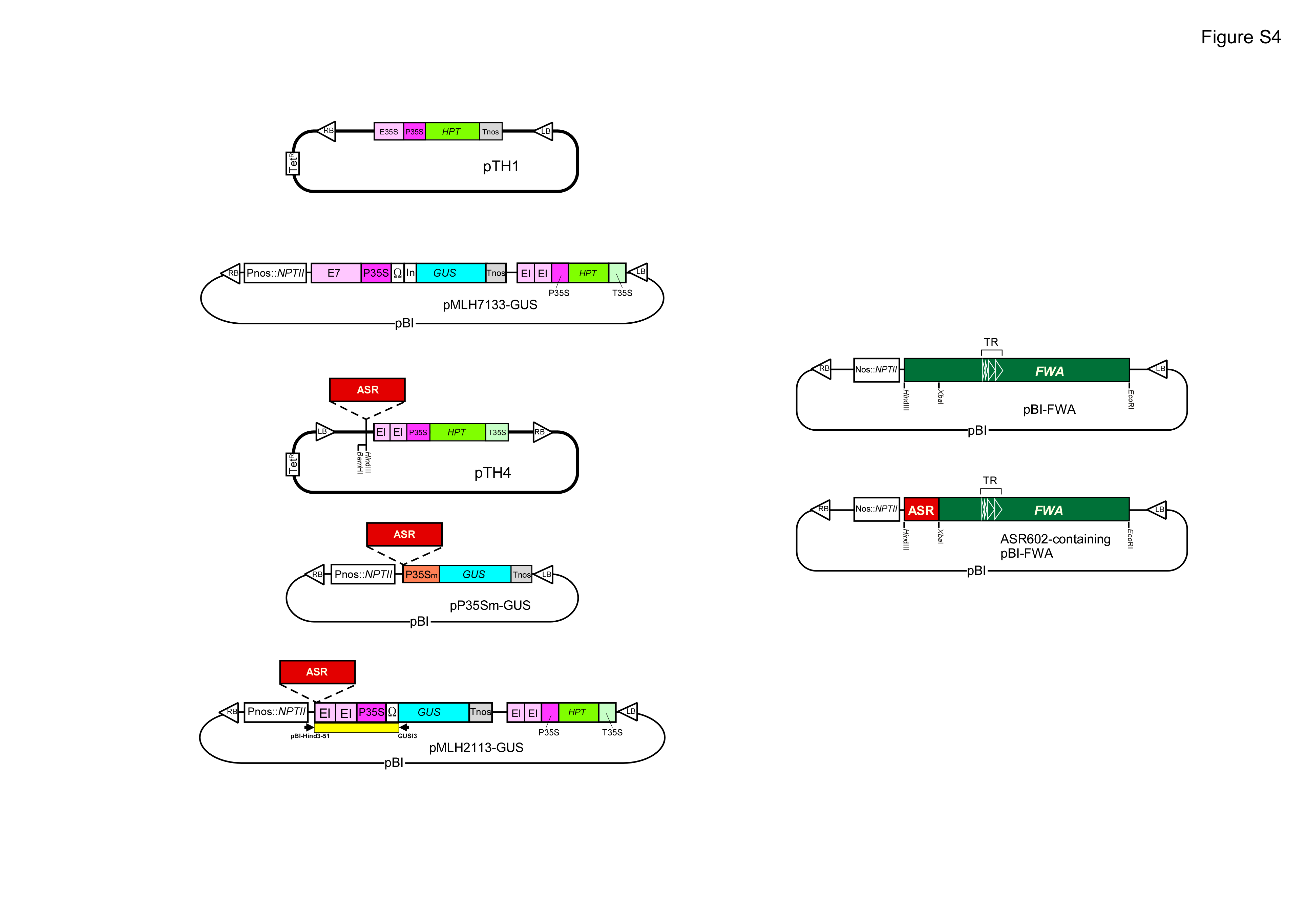

Supplement: Figure S4 — Binary vector constructs used for this study (see also Materials and Methods). pTH1 was used to supertransform TGS plants for selection of plants showing trans-TGS activity. This vector contains the CaMV 35S enhancer region (E35S) and promoter region (P35S) of pBI121, followed by HPT. RB and LB, right and left borders of T-DNA of Agrobacterium tumefaciens Ti plasmid, respectively. E35S, 5′-upstream sequence of CaMV 35S promoter (–940 to –90). P35S, 5′-upstream sequence of CaMV 35S promoter (–90 to –1). HPT, hygromycin phosphotransferase gene (a selectable marker). Tnos, polyadenylation signal of the nopaline synthase gene (nos) in the Ti plasmid. TetR, a tetracycline-resistance marker gene. pMLH7133-GUS was used as a second CaMV 35S promoter-driven construct to confirm trans-TGS activity of the trans-TGS plant M66-9. The GUS expression cassette in this construct contains seven copies of the CaMV 35S enhancer (E7) and the P35S. Pnos:: NPTII, nos promoter-driven neomycin phosphotransferase gene (a selectable marker) that confers resistance to kanamycin. E7, 5′-upstream sequence of CaMV 35S promoter (–940 to –290) and (–290 to –90) x 7. Ω, 5′-untranslated sequence of tobacco mosaic virus. In, first intron of a phaseolin gene. GUS, β-glucuronidase gene (a reporter gene). T35S, polyadenylation signal of the CaMV 35S transcript. pTH4 was used to construct the genomic library of L. japonicus for ASR screening. This vector contains two copies of the CaMV 35S enhancer (El) and the P35S. El, 5′-upstream sequence of CaMV 35S promoter (–419 to –90). pP35Sm-GUS was used to examine whether an ASR candidate has enhancer activity. This construct harbors the –46 CaMV 35S minimal promoter region. pMLH2113-GUS was used to supertransform the LUC tobacco plant (Figure 3A, NW7-24-4). This construct carries two copies of El and the P35S. The yellow bar depicts the region where the methylation status was analyzed by methylation-sensitive restriction enzyme-coupled PCR assay (Figure 3C). Tw [file pone.0054670.s004.tif]

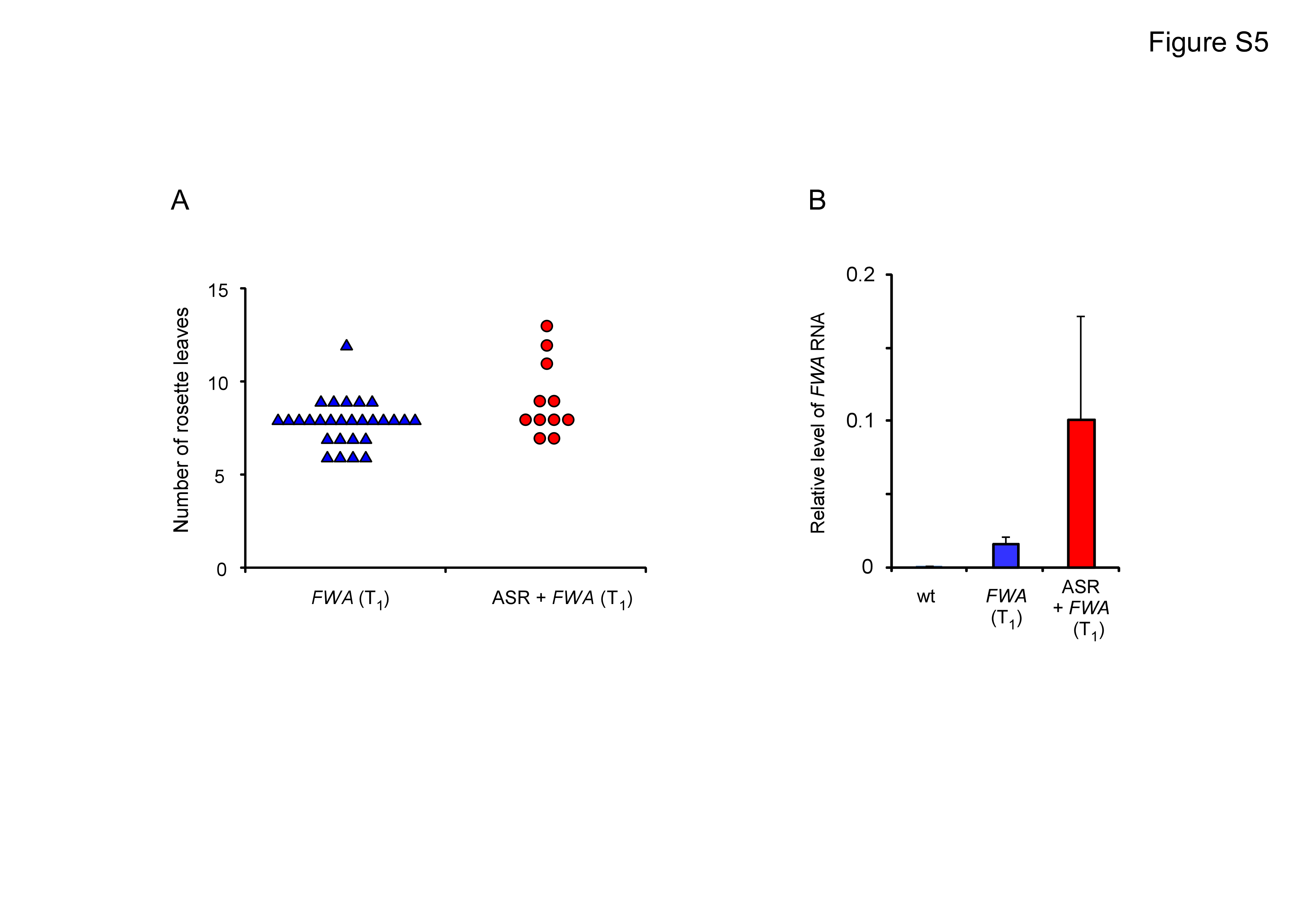

Supplement: Figure S5 — Flowering time and FWA expression of T1 generation derived from the T0 transformants, “ FWA ” and “ASR+ FWA ”, shown in Figure 3 . (A) Flowering time. Each triangle/dot depicts a T1 plant individual. T1 seeds were collected in bulk from each T0 generation group. (B) Quantitative real-time polymerase chain reaction analysis of FWA RNA in leaf of T1 plant. Methods of the PCR analysis was described elsewhere [42]. Prime sets used were shown in Table S1. wt (Col-0), n = 3; FWA (T1), n = 12; ASR+FWA (T1), n = 11; n, number of plant individuals. Mean values are given. Error bars denote ± s.e.m. (TIF) [file pone.0054670.s005.tif]
