# Supplementary material for: DNA Elements Reducing Transcriptional Gene Silencing Revealed by a Novel Screening Strategy
Source: PLoS One. 2013 Jan 30;8(1):e54670. doi: 10.1371/journal.pone.0054670 (PMC3559876; doi:10.1371/journal.pone.0054670)
Supplement: Table S1 — Oligonucleotide primers used in this study. (DOC) [file pone.0054670.s006.doc]

**Table S1. Oligonucleotide primers used in this study**

| **Primer name** | **Primer sequence** | **PCR products and features** |
| --- | --- | --- |
| BL31 | AAT ATG GGC AGC GAA GGC C | Anti-silencing regions (ASRs) from plants supertransformed with the pTH4 genomic library of *Lotus japonicus* |
| P35-31 | CGT CAT CCC TTA CGT CAG TGG AGA T | Anti-silencing regions (ASRs) from plants supertransformed with the pTH4 genomic library of *Lotus japonicus* |
| pBI-Hind3-51 | ATG ACC ATG ATT ACG CCA AGC | Methylated status of a region in the *CaMV* 35S enhancer/promoter of pMLH2113-GUS |
| GUSI3 | TAC GAA TAT CTG CAT CGG CG | Methylated status of a region in the *CaMV* 35S enhancer/promoter of pMLH2113-GUS |
| GUSI51 | GCA GGG AGG CAA ACA ATG AA | Copy number of *GUS* and *LUC* genes in supertransformed *LUC*-transgenic plants (from *GUS* or *LUC* to the *nos* terminator) |
| LUCI51 | GTA ATT TTG TAA TTG TGG GTC AC | Copy number of *GUS* and *LUC* genes in supertransformed *LUC*-transgenic plants (from *GUS* or *LUC* to the *nos* terminator) |
| Tnos Yomeru | TGT TTG AAC GAT CGG GGA AAT | Copy number of *GUS* and *LUC* genes in supertransformed *LUC*-transgenic plants (from *GUS* or *LUC* to the *nos* terminator) |
| UP FWA51 | TTA AAA GCT TAG TAA ATC ATT GTG GCG ACA CTT TTT TTC GTC TC | *FWA* gene from *Arabidopsis thaliana* genome DNA (5.5kb between positions 13037026 and 13042519 in GenBank accession NC_003075). AAGCTT: inserted *Hin*dIII site |
| FWA32 | TTA AGA ATT CTG GAA TGC ATT TTT CAC CTG ATA TAG TG | *FWA* gene from *Arabidopsis thaliana* genome DNA (5.5kb between positions 13037026 and 13042519 in GenBank accession NC_003075). GAATTC: an endogenous *Eco*RI site |
| AtFWA3UTR51 | CCG TTG TTC CGC AAG AAA TTC G | Quantitative real-time PCR of FWA RNA in leaf of T1 plant |
| AtFWA3UTR31 | AAG CAA GAA ATA TCC AGC AAA CAG ATC | Quantitative real-time PCR of FWA RNA in leaf of T1 plant |
| AtActin2F | GGT AAC ATT GTG CTC AGT GGT GG | Quantitative real-time PCR of control RNA in leaf of T1 plant |
| AtActin2R | GGT GCA ACG ACC TTA ATC TTC AT | Quantitative real-time PCR of control RNA in leaf of T1 plant |
