# Supplementary material for: DNA Elements Reducing Transcriptional Gene Silencing Revealed by a Novel Screening Strategy
Source: PLoS One. 2013 Jan 30;8(1):e54670. doi: 10.1371/journal.pone.0054670 (PMC3559876; doi:10.1371/journal.pone.0054670)
Supplement: Table S2 — Anti-silencing regions (ASR) do not have enhancer activity. (DOC) [file pone.0054670.s007.doc]

**Table S2. Anti-silencing regions (ASR) do not have enhancer activity**

|  | **GUS activity** | |  |
| --- | --- | --- | --- |
| **Construct** | **Average** | **SE** | ***P* value** |
| Control | 0.77 | 0.18 | n/a |
| ASR102 | 0.29 | 0.01 | 1.03 x 10^-2^ * |
| ASR501 | 0.88 | 0.29 | 9.99 x 10^-1^ |
| ASR602 | 0.57 | 0.13 | 9.97 x 10^-1^ |

To examine if ASRs have enhancer activity, the pP35Sm-GUS (without ASR) and the ASR-containing derivative constructs were transformed into cultured cells of *Nicotiana tabacum* (cv. Samsun NN) (Figure S4). Twenty transformants for each construct were examined. The data were transformed according to a standard textbook of biostatistics [30] and then Dunnett’s test was used to compare each ASR with the control [43,44]. GUS activity, 4-MU nmol mg protein^–1^ min^–1^. 4-MU, 4-methylumbelliferone.

* 0.01 < *P* < 0.05
