# Supplementary material for: DNA Elements Reducing Transcriptional Gene Silencing Revealed by a Novel Screening Strategy
Source: PLoS One. 2013 Jan 30;8(1):e54670. doi: 10.1371/journal.pone.0054670 (PMC3559876; doi:10.1371/journal.pone.0054670)
Supplement: Table S3 — Pearson’s correlation between the P35S::GUS and the P35S::LUC expression levels in Figure 2D . (DOC) [file pone.0054670.s008.doc]

**Table S3. Pearson’s correlation between the P35S::*GUS* and the P35S::*LUC* expression levels in Figure 2D**

| **Supertransformants^1^** | **n** | **Pearson's *r*** | ***P* value** |
| --- | --- | --- | --- |
| CST/Total | 28 | 0.44 | 1.9 x 10^-2^ * |
| ASR602 ST/Total | 22 | 0.37 | 9.4 x 10^-2^ |
| CST/1 copy | 13 | 0.42 | 1.5 x 10^-1^ |
| ASR602 ST/1 copy | 12 | 0.02 | 9.6 x 10^-1^ |

^1^ See Figure 2D for details

* 0.01 < *P* < 0.05
