# Supplementary material for: DNA Elements Reducing Transcriptional Gene Silencing Revealed by a Novel Screening Strategy
Source: PLoS One. 2013 Jan 30;8(1):e54670. doi: 10.1371/journal.pone.0054670 (PMC3559876; doi:10.1371/journal.pone.0054670)
Supplement: Text S1 — Primary structure of three ASR candidates. (References for supporting information are compiled as “Supporting Information References” on the last page of the.doc file Text S1. (DOC) [file pone.0054670.s009.doc]

**Text S1. Primary structure of three ASR candidates.**

The nucleotide sequences of the three ASR candidates were submitted (on 12 July 2011) as queries using the default BLAST program of GenBank (http://blast.ncbi.nlm.nih.gov/Blast.cgi). There was no sequence similarity between these ASR candidates (E-values > 0.009 by “bl2seq” BLASTN).

ASR102 (ca. 3.0 Kbp) showed highly significant similarity to a genomic sequence of *L. japonicus* (AP010447.1) (maximum score = 5229, query coverage = 100%, E-value = 0, maximum identity = 98% by BLASTN). Also, ASR102 showed marked similarity to six ESTs (E-values < 10^-13^) and to 52 hypothetical proteins (E-values < 10^-40^ by BLASTX). A deduced amino acid sequence suggested that ASR102 included a retropepsin-like domain (also called as a retroviral aspartyl protease domain; Pfam00077) (E-value = 1x10^-8^ by NCBI Conserved Domain Search; http://www.ncbi.nlm.nih.gov/Structure/cdd/wrpsb.cgi). Further analyses by BLASTX at the peptidase database MEROPS, (http://merops.sanger.ac.uk/cgi-bin/blast/submitblast/merops/advanced) revealed that ASR102 had an aspartic protease domain classified as belonging to the “cauliflower mosaic virus-type peptidase” (A3) family by MEROPS (E-value = 4x10^-2^), implying that the ASR102 sequence was originally derived from a *Caulimoviridae* pararetrovirus. Indeed, amino acid sequences of reverse transcriptase and RNase H in *Caulimoviridae* viruses were well aligned to deduced amino acid sequences of the *Lotus* genomic sequences that contain ASR102 itself and the most similar sequence to ASR102.

ASR501 (ca. 0.3 Kbp) showed highly significant similarity to a genomic sequence of *L. japonicus* (AP010439.1) (Maximum score = 553, query coverage = 100%, E-value = 10^-153^, maximum identity = 99% by BLASTN). ASR501 also showed marked similarity to 18 ESTs (E-values < 10^-7^ by BLASTN) and to several “with no lysine” kinases (E-values < 8x10^-4^ by BLASTX).

When the ASR602 sequence (171 bp) was used as a query in BLASTN searches against the non-redundant nucleotide database of GenBank (http://blast.ncbi.nlm.nih.gov/Blast.cgi), the query sequence yielded a large number of hits (169 non-redundant entries, E-values < 10^-11^) from *L. japonicus*, but no hits from any other organisms (E-values > 0.054); when the query sequence was used against the EST databases of GenBank, the search yielded three *Lotus* ESTs (E-values < 10^-21^); the query sequence yielded no hits from any other organisms (E-values > 10^-4^) in any of the remaining 13 databases of BLASTN in GenBank. ASR602 itself has not been registered in any sequence database, while sequences similar to ASR602 were abundant and well-conserved in the *L. japonicus* genome; similar sequences were found frequently in independent contigs of *L. japonicus* that have been assigned to chromosomes (http://www.kazusa.or.jp/lotus/index.html) (Figure S3). Manual inspection of the contigs of *L. japonicus* consisting of non-redundant entries suggested that sequences similar to ASR602 were portions from members of a retrotransposon family (Figures 2 and S2), whose long terminal repeats (LTRs) were species-specific (1.2 Kbp). A BlastX search of genomic units sandwiched between the LTRs revealed that all of the units examined contained conserved protein domains in retrotransposons of the Ty1-*copia* family (Figures 2 and S2). The units were also flanked by target site duplications of 5-base sequences that vary with location. These results suggested that the units originated from a retrotransposon of the Ty1/*copia* family.

Next, to see if ASR602 includes sequence motif(s) of scaffold/matrix attachment regions (S/MARs), we used three tools for identifying putative S/MARs: MAR-Wiz [45], Marscan [46] and Chrclass [47]. MAR-Wiz and Marscan were accessed at http://genomecluster.secs.oakland.edu/marwiz/ and EMBOSS, http://emboss.bioinformatics.nl/cgi-bin/emboss/marscan, respectively. Chrclass was downloaded from http://ftp.bionet.nsc.ru/pub/biology/chrclass/chrclass2.zip (in this version, the matrix recognition signature criterion used in Marscan is also implemented). The sequence of ASR602 (171 bp) was too short for analysis using these tools. As the next best alternative, we analyzed a “padded” sequence in which 1 Kbp monomer sequences of A, T, G, or C were added at both ends of the ASR602 sequence. No S/MAR was detected in the ASR602 sequence with either tool. We then analyzed eight genomic sequences, including eight ASLs (ASL1–ASL8) that harbor the most ASR602-like sequences (Figure 2). No S/MAR was detected in these eight ASR602-like sequences.

**Supporting Information References**

43. Dunnett CW (1995) A multiple comparison procedure for comparing several treatments with a control. J Am Stat Assoc 50: 1096–1121.

44. Dunnett CW (1964) New tables for multiple comparisons with a control. Biometrics 20: 482–491.

45. Singh GB, Kramer JA, Krawetz SA (1997) Mathematical model to predict regions of chromatin attachment to the nuclear matrix. Nucleic Acids Res 25: 1419-1425.

46. van Drunen CM, Sewalt RG, Oosterling RW, Weisbeek PJ, Smeekens SC, et al. (1999) A bipartite sequence element associated with matrix/scaffold attachment regions. Nucleic Acids Res 27: 2924-2930.

47. Glazko GV, Rogozin IB, Glazkov MV (2001) Comparative study and prediction of DNA fragments associated with various elements of the nuclear matrix. Biochem Biophys Acta 1517: 351-364.
